# Supplementary material for: Antioxidant Potential and Enhancement of Bioactive Metabolite Production in In Vitro Cultures of Scutellaria lateriflora L. by Biotechnological Methods
Source: Molecules. 2022 Feb 8;27(3):1140. doi: 10.3390/molecules27031140 (PMC8839037; doi:10.3390/molecules27031140)
Supplement: Supplementary file 1 [file molecules-27-01140-s001.zip › fig S1.pdf]

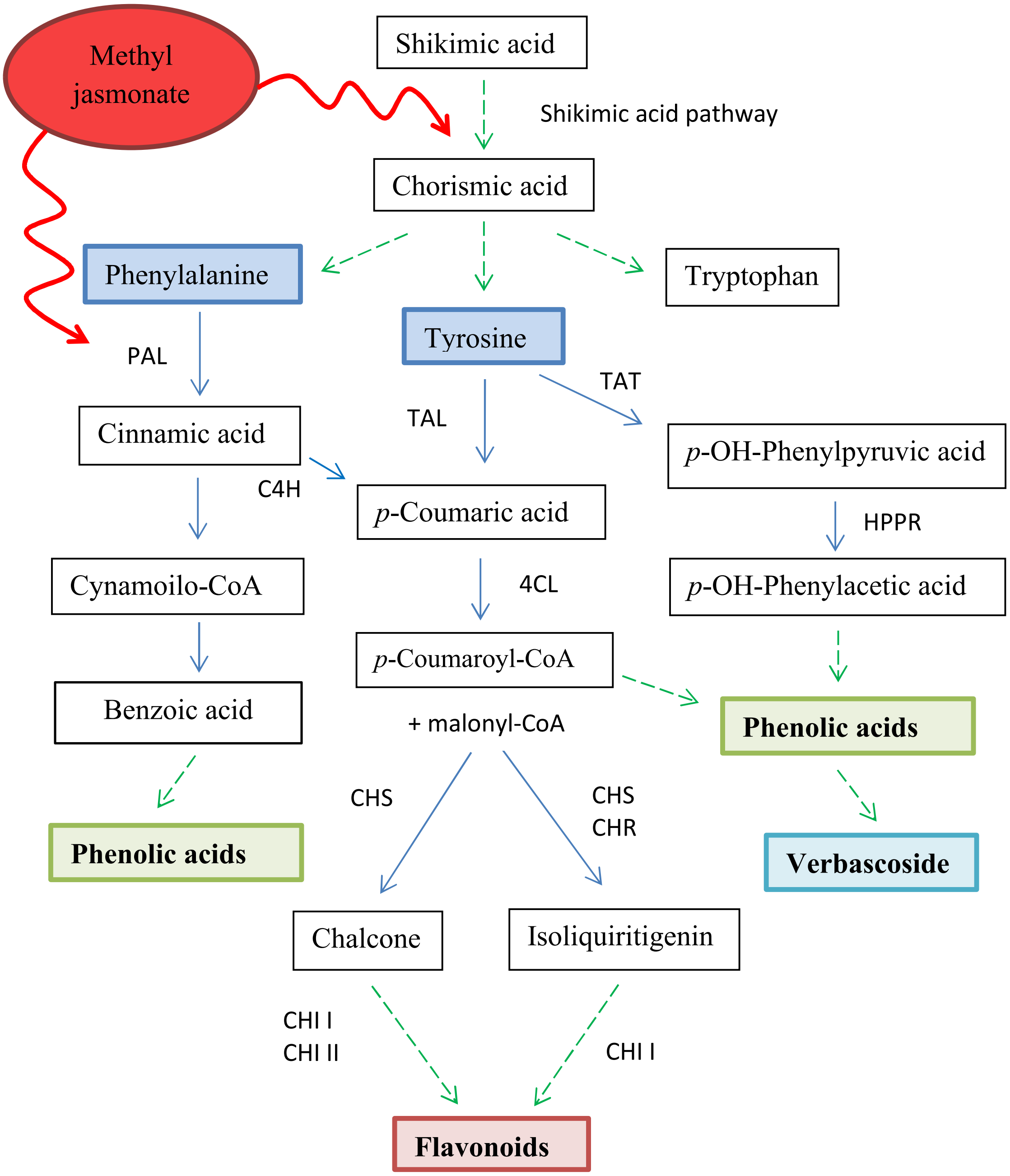

Figure S1. Phenolic compounds biosynthesis pathways.

Abbreviations:
C4H - cinnamate-4-hydroxylase; CHI - chalcone isomerase; CHR – chalcone reductase; CHS - chalcone synthase; 4CL - 4-coumaroyl:CoA-ligase; HPPR - 4-hydroxyphenylpyruvate reductase; PAL - phenylalanine ammonia-lyase; TAL - tyrosineammonia-lyase; TAT – tyrosine aminotransferase;
