# Supplementary material for: Antioxidant Potential and Enhancement of Bioactive Metabolite Production in In Vitro Cultures of Scutellaria lateriflora L. by Biotechnological Methods
Source: Molecules. 2022 Feb 8;27(3):1140. doi: 10.3390/molecules27031140 (PMC8839037; doi:10.3390/molecules27031140)
Supplement: Supplementary file 1 [file molecules-27-01140-s001.zip › table SF 1-2a.pdf]

Table S1 Content of estimated metabolites in agitated microshoot in vitro cultures of *S. lateriflora* grown on LS medium supplemented with 1.0 mg/L BA and 1.0 NAA mg/L after administering different concentrations of biosynthetic precursors (phenylalanine and tyrosine) and elicitor (methyl jasmonate) collected 3 days after supplementation. Mean ± SD [mg/100 g DW].

Feeding/eliciting		Baicalein	Baicalin	Wogonin	Wogonoside	Scutellarin	Oroxylin A	Total flavonoids	Verbascoside
Control		171.557 ± 12.516	445.713 ± 20.473	689.250 ± 24.371	424.741 ± 11.473	4.065 ± 0.927	4.620 ± 0.788	1739.947 ± 70.548	311.197 ± 40.548
Phenylalanine [g/L]	1.0	334.258 ± 18.753	575.217 ± 18.189	764.828 ± 50.536	827.073 ± 58.224	72.340 ± 13.252	17.224 ± 1.534	2590.941 ± 160.489	463.414 ± 60.489
	1.5	533.084 ± 21.941	698.826 ± 35.761	808.929 ± 64.103	935.389 ± 67.641	30.648 ± 9.536	34.814 ± 2.934	3041.690 ± 201.914	469.127 ± 91.914
	2.0	435.692 ± 32.555	590.140 ± 56.988	777.430 ± 65.114	905.162 ± 78.460	27.527 ± 2.972	30.873 ± 5.557	2766.825 ± 241.644	379.043 ± 41.644
	2.5	330.399 ± 27.374	449.045 ± 40.104	687.909 ± 37.977	885.060 ± 43.051	28.722 ± 5.183	32.812 ± 3.366	2413.946 ± 157.056	346.063 ± 57.056
Tyrosine [g/L]	1.0	304.790 ± 13.156	431.531 ± 19.470	747.038 ± 15.450	407.018 ± 21.356	11.714 ± 2.075	29.693 ± 4.480	1931.784 ± 75.987	299.480 ± 75.987
	1.5	315.664 ± 28.169	442.068 ± 20.460	749.526 ± 15.489	388.409 ± 22.555	14.205 ± 1.264	18.889 ± 1.150	1928.760 ± 89.087	222.880 ± 49.087
	2.0	199.789 ± 16.897	196.472 ± 16.015	546.742 ±9.012	310.049 ± 10.132	8.810 ± 0.188	22.051 ± 5.243	1283.913 ± 57.489	166.752 ± 57.489
	2.5	187.658 ± 13.839	255.948 ± 44.914	484.239 ± 66.116	220.618 ± 43.480	1.910 ± 0.079	25.742 ± 2.520	1176.114 ± 170.949	171.135 ± 17.949
Methyl jasmonate [µM]	10	351.450 ± 41.801	552.318 ± 23.506	649.821 ± 21.065	469.450 ± 23.055	12.588 ± 4.015	23.924 ± 1.454	2059.550 ± 114.897	381.523 ± 14.897
	50	298.456 ± 22.566	546.947 ± 26.662	676.638 ± 25.215	421.951 ± 56.217	25.840 ± 6.515	37.418 ± 2.325	2007.250 ± 139.498	133.698 ± 39.498
	100	221.490 ± 16.340	290.315 ± 17.305	149.515 ± 26.980	281.021 ± 18.593	1.098 ± 0.161	33.121 ± 5.111	976.560 ± 84.489	20.696 ± 8.489
Phenylalanine [g/L] + methyl jasmonate 50µM	1.0	301.654 ± 39.887	571.818 ± 59.332	709.401 ± 86.104	527.879 ± 60.244	12.122 ± 1.469	33.493 ± 2.452	2156.366 ± 249.489	141.078 ± 24.489
	1.5	527.640 ± 57.302	610.602 ± 78.309	745.436 ± 77.691	602.067 ± 85.539	17.999 ± 5.157	22.153 ± 3.881	2525.897 ± 307.879	160.597 ± 37.879
	2.0	487.657 ± 57.049	575.362 ± 69.760	608.223 ± 77.489	590.686 ± 66.980	15.461 ± 3.549	47.607 ± 4.670	2324.996 ± 279.498	142.153 ± 29.498
	2.5	302.749 ± 22.796	561.104 ± 42.587	602.598 ± 48.517	512.708 ± 49.738	10.038 ± 1.163	0.134 ± 0.098	1989.330 ± 164.899	120.270 ± 16.899
Tyrosine [g/L] + methyl jasmonate 50 µM	1.0	257.450 ± 8.652	507.194 ± 11.708	659.016 ± 10.974	442.457 ± 18.941	17.452 ± 1.758	22.427 ± 3.944	1905.997 ± 55.977	21.176 ± 5.977
	1.5	212.687 ± 13.469	344.997 ± 30.540	356.136 ± 65.189	381.224 ± 54.752	10.038 ± 0.915	27.505 ± 2.540	1332.587 ± 167.405	25.460 ± 7.405
	2.0	105.468 ± 17.619	140.292 ± 15.040	120.460 ± 39.044	360.621 ± 40.610	3.048 ± ± 0.652	0.372 ± 0.014	730.261 ± 112.979	17.156 ± 2.979
	2.5	88.973 ± 7.140	97.026 ± 21.534	121.561 ± 33.081	220.927 ± 35.617	0.527 ± 0.058	4.309 ± 1.052	533.322 ± 98.484	10.175 ± 2.484

Table S2 Content of estimated metabolites in agitated microshoot in vitro cultures of *S. lateriflora* grown on LS medium supplemented with 1.0 mg/L BA and 1.0 NAA mg/L after administering different concentrations of biosynthetic precursors (phenylalanine and tyrosine) and elicitor (methyl jasmonate) collected 7 days after supplementation. Mean ± SD [mg/100 g DW].

Feeding/eliciting		Baicalein	Baicalin	Wogonin	Wogonoside	Scutellarin	Oroxylin A	Total flavonoids	Verbascoside
Control		162.414 ± 26.104	378.595 ± 26.606	666.088 ± 71.518	469.987 ± 59.996	2.539 ± 0.940	0.214 ± 0.023	1679.837 ± 185.187	267.327 ± 15.187
Phenylalanine [g/L]	1.0	421.423 ± 34.919	686.268 ± 45.593	825.584 ± 78.669	928.457 ± 112.633	64.221 ± 17.094	38.498 ± 8.280	2964.451 ± 297.188	398.597 ± 27.188
	1.5	614.788 ± 38.279	928.948 ± 55.794	964.501 ± 49.668	1138.451 ± 64.939	68.295 ± 4.980	49.898 ± 3.828	3764.881 ± 217.489	474.795 ± 27.489
	2.0	516.970 ± 25.841	988.086 ± 56.083	875.558 ± 43.162	1148.771 ± 50.697	60.817 ± 6.254	53.653 ± 4.450	3643.854 ± 186.487	329.667 ± 36.487
	2.5	596.308 ± 23.850	760.204 ± 11.426	727.718 ± 21.986	910.827 ± 13.158	62.108 ± 7.751	30.143 ± 6.616	3087.308 ± 84.786	327.734 ± 34.786
Tyrosine [g/L]	1.0	157.460 ± 18.653	419.209 ± 21.780	655.544 ± 30.779	516.879 ± 56.240	9.321 ± 1.797	41.057 ± 8.250	1799.470 ± 137.499	263.961 ± 37.499
	1.5	162.457 ± 49.878	284.688 ± 45.369	270.264 ± 79.089	485.827 ± 131.150	3.751 ± 0.678	41.758 ± 1.730	1248.745 ± 307.894	210.926 ± 37.894
	2.0	113.764 ± 23.568	113.103 ± 16.217	233.897 ± 45.127	300.340 ± 45.344	0.084 ± 0.007	21.818 ± 1.459	783.007 ± 131.722	145.963 ± 31.722
	2.5	98.154 ± 22.671	111.861 ± 35.212	232.868 ± 68.849	250.713 ± 94.841	0.041 ± 0.009	19.392 ± 6.389	713.028 ± 227.97	128.312 ± 27.970
Methyl jasmonate [μM]	10	254.440 ± 27.292	474.399 ± 49.675	657.090 ± 67.793	328.520 ± 43.090	5.292 ± 1.872	43.708 ± 3.768	1763.448 ± 193.489	121.553 ± 19.489
	50	300.486 ± 40.901	427.820 ± 20.453	676.857 ± 55.040	365.481 ± 17.298	10.087 ± 2.591	34.146 ± 1.175	1814.878 ± 137.456	52.582 ± 13.456
	100	190.564 ± 37.074	165.356 ± 28.380	183.494 ± 75.055	218.359 ± 85.208	0.040 ± 0.007	4.444 ± 0.632	762.256 ± 226.354	41.891 ± 6.354
Phenylalanine [g/L] + methyl jasmonate 50μM	1.0	325.480 ± 21.628	543.161 ± 21.289	698.489 ± 19.103	601.435 ± 28.710	18.946 ± 4.985	24.858 ± 1.442	2212.369 ± 97.156	170.694 ± 17.156
	1.5	541.608 ± 36.215	630.484 ± 17.532	782.986 ± 29.100	620.747 ± 30.154	18.046 ± 3.460	23.002 ± 4.107	2616.874 ± 120.568	142.323 ± 12.568
	2.0	502.489 ± 58.489	623.827 ± 75.770	657.050 ± 90.489	590.761 ± 82.540	15.346 ± 2.057	25.006 ± 7.145	2414.479 ± 316.489	28.368 ± 6.489
	2.5	365.978 ± 34.335	525.029 ± 57.465	547.899 ± 74.527	416.988 ± 86.413	10.181 ± 1.152	16.730 ± 2.263	1882.805 ± 256.156	117.389 ± 25.156
Tyrosine [g/L] + methyl jasmonate 50 μM	1.0	264.457 ± 31.590	411.619 ± 41.777	599.387 ± 51.315	479.433 ± 51.441	9.041 ± 0.800	14.562 ±.2.246	1778.499 ± 179.168	24.220 ± 1.168
	1.5	170.544 ± 20.489	390.586 ± 23.498	415.977 ± 44.241	403.332 ± 45.150	10.042 ± 1.025	8.812 ± 0.489	1399.293 ± 134.892	44.068 ± 4.892
	2.0	120.560 ± 14.679	114.740 ± 17.550	330.750 ± 35.804	321.291 ± 43.159	3.693 ± 0.054	6.562 ± 1.250	897.595 ± 112.496	38.745 ± 12.496
	2.5	74.979 ± 9.588	64.506 ± 8.850	280.974 ± 24.238	204.285 ± 35.218	5.038 ± 0.727	7.983 ± 0.843	637.766 ± 89.464	43.942 ± 8.464
