# Supplementary figures and images for: Antioxidant Potential and Enhancement of Bioactive Metabolite Production in In Vitro Cultures of Scutellaria lateriflora L. by Biotechnological Methods
Source: Molecules. 2022 Feb 8;27(3):1140. doi: 10.3390/molecules27031140 (PMC8839037; doi:10.3390/molecules27031140)

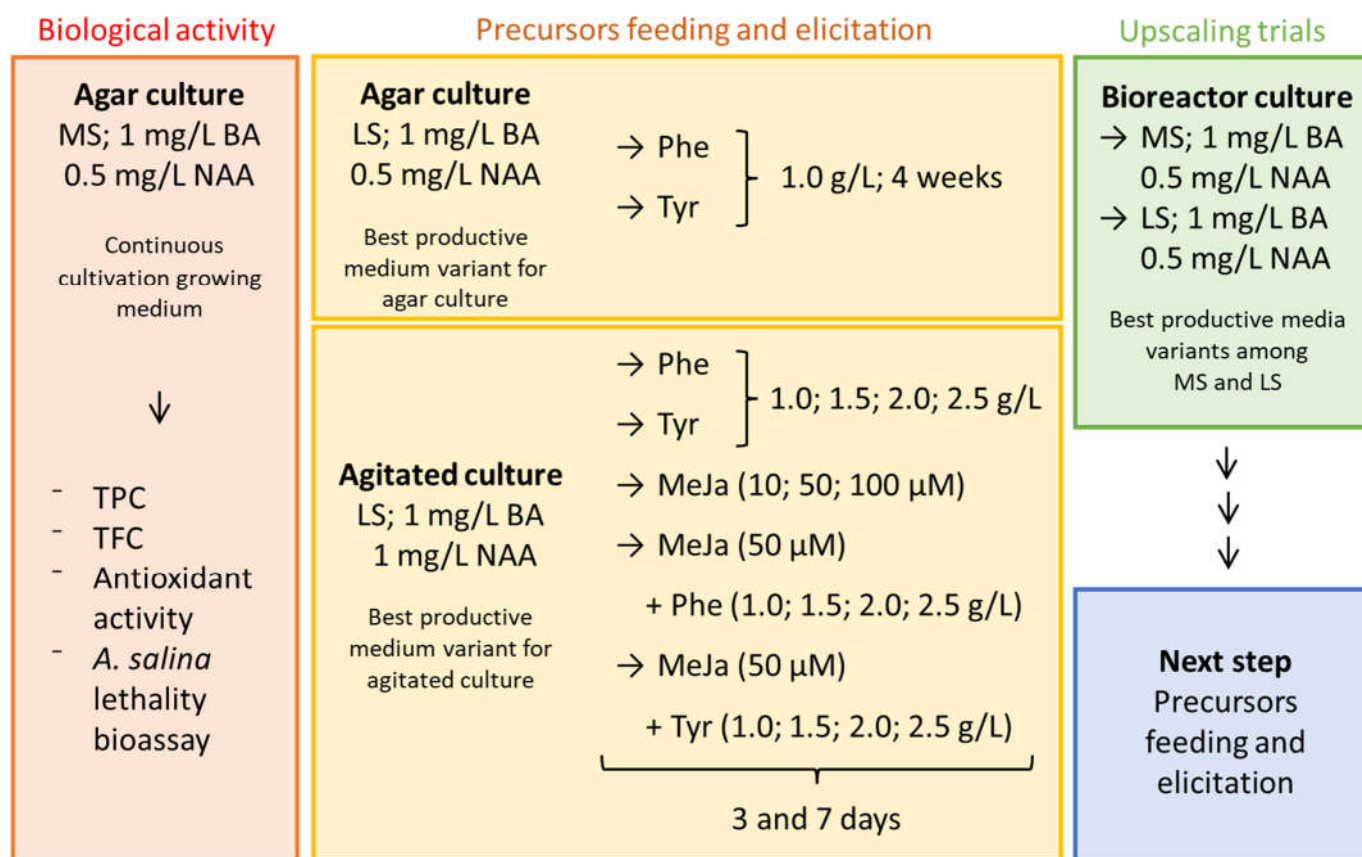

Figure S2. *Scutellaria lateriflora* microshoot cultures. Diagram of the experimental process.

Supplement: Supplementary file 1 [file molecules-27-01140-s001.zip › fig S2.pdf]
